# Supplementary material for: Transcriptome Responses in Medicago sativa (Alfalfa) Associated with Regrowth Process in Different Grazing Intensities
Source: Plants (Basel). 2024 Sep 30;13(19):2738. doi: 10.3390/plants13192738 (PMC11479129; doi:10.3390/plants13192738)
Supplement: Supplementary file 1 [file plants-13-02738-s001.zip › Supplyment Figures.pdf]

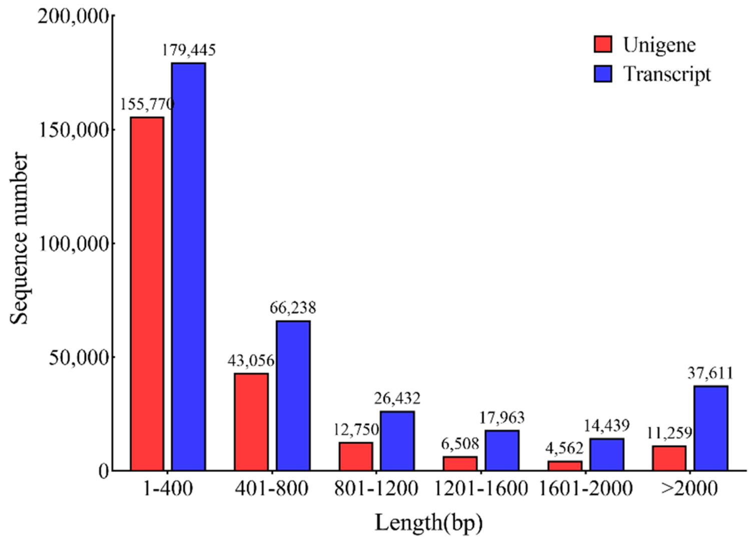

Figure S1. Transcript and unigene length distribution.

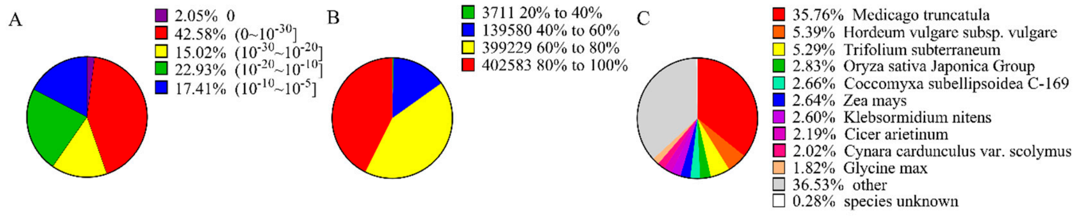

Figure S2. Performing unigene homology searches in the Nr database: A. The proportional frequency of the E-value distribution. B. The proportional frequency of the sequence similarity distribution. C. The proportional species distribution of M. sativa. among other plant species.

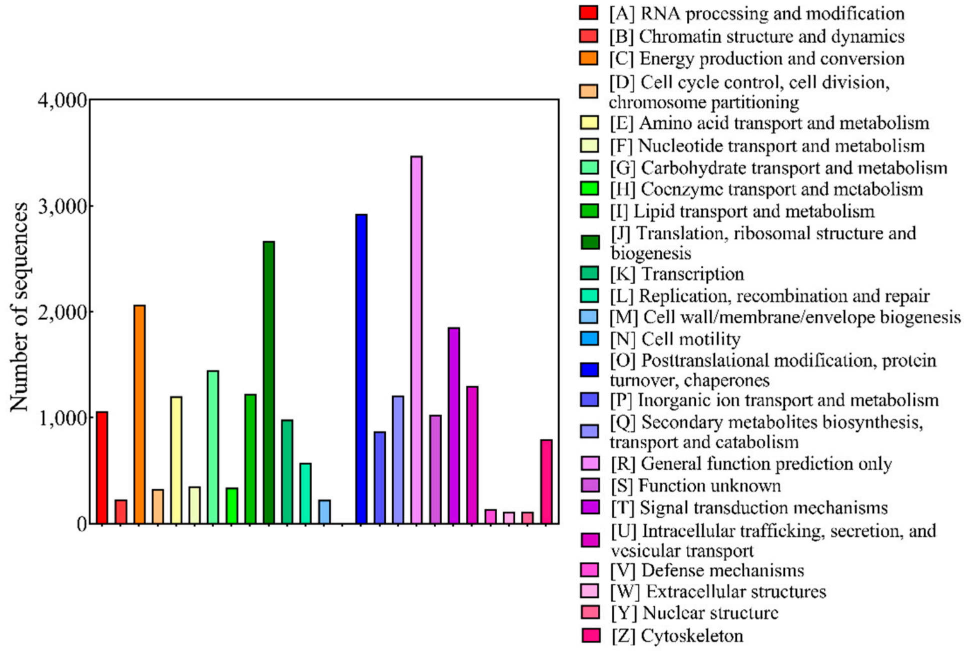

Figure S3. KOG function classification of unigenes for *M. sativa.* The left y-axis indicates the number of unigenes; the letters on the x-axis represent different KOG categories.
